# Supplementary material for: Using B isotopes and B/Ca in corals from low saturation springs to constrain calcification mechanisms
Source: Nat Commun. 2019 Aug 8;10:3580. doi: 10.1038/s41467-019-11519-9 (PMC6687739; doi:10.1038/s41467-019-11519-9)
Supplement: Supplementary file 1 — Supplementary Information [file 41467_2019_11519_MOESM1_ESM.docx]

**Supplementary Information**

**Using B isotopes and B/Ca in corals from low saturation springs to constrain calcification mechanisms**

Wall et al.

**Supplementary Figures**


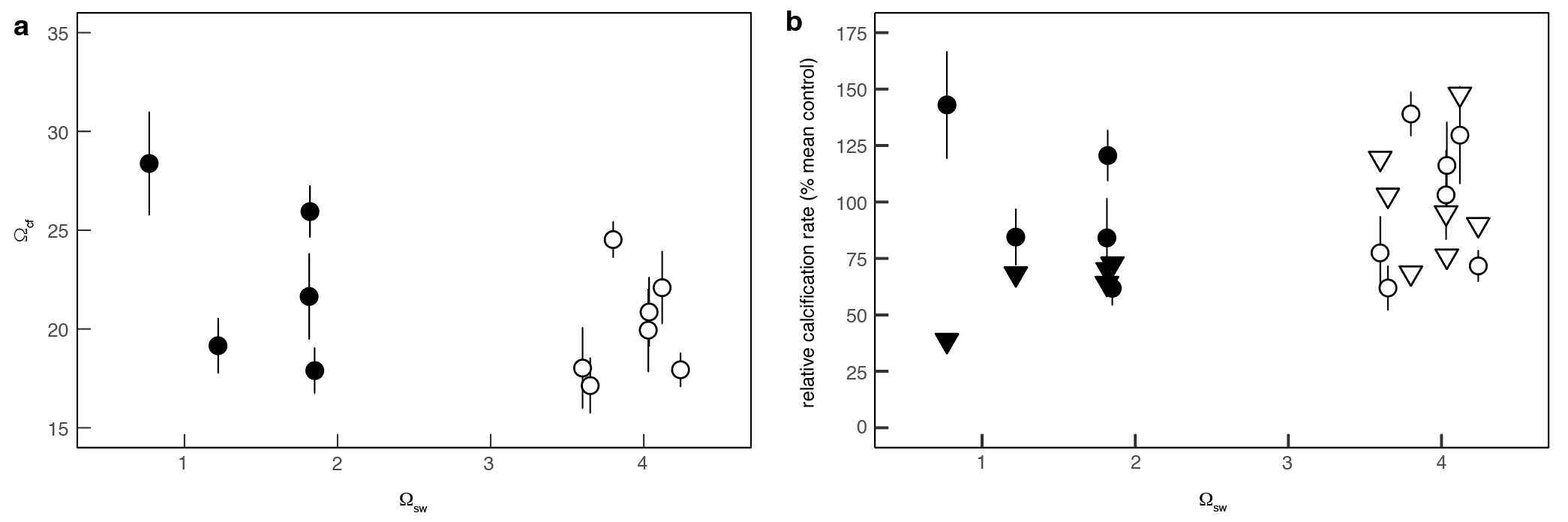


**Supplementary Figure 1:** **Growth response modelled for *Porites astreoides* corals**. The modelled growth response displays relative changes in calcification rate (relative calcification rate = mean control/individual colony). Calcification rates were calculated following the IpHRAC model ^1^ (internal pH regulation and abiotic calcification: Calcification =k*(Ω_cf_-1)^n^ ) with Ω_cf_ was calculated from the average internal pH_cf_ of individual colonies and a dissolved inorganic carbon (DIC) concentrations that was twice the concentration seawater DIC (DIC_cf_ = 2 x DIC_sw_): in (a) dependent variable Ω_cf_ is based on 2xDIC_sw_ and pH_cf_ and (b) depicts the respective calculated calcification rates. Circles represent values for each individual coral colony (mean ± ci). Filled and non-filled symbols denotes the different locations: filled are the centers of the ojos with lower Ω_sw_ and non-filled the control high Ω_sw_ site. Individual values are mean ± 95%-CI.


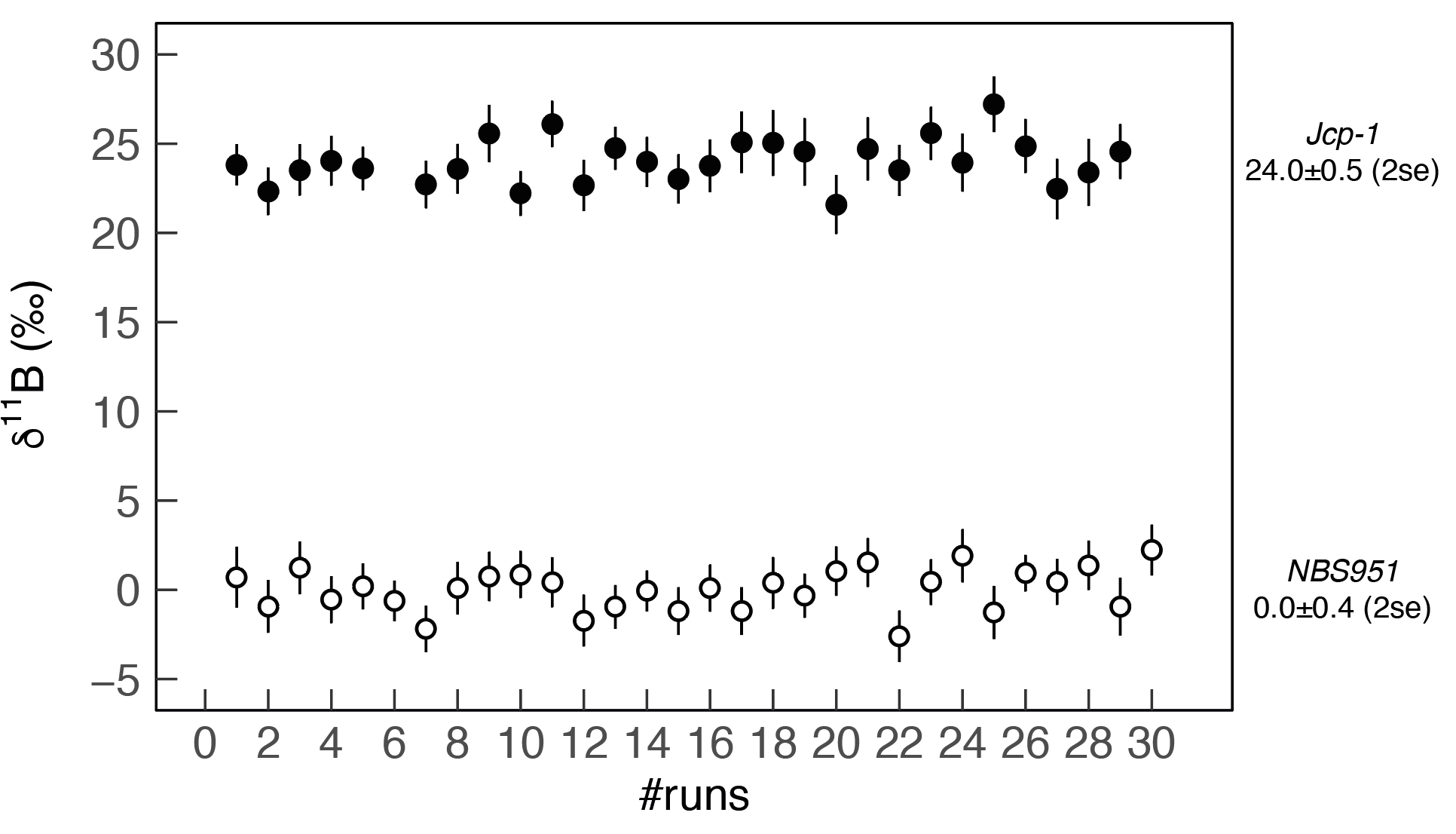


**Supplementary Figure 2:** **Repeated analyses of Jcp-1 and boric acid standard NBS951*.*** Individual runs of laser ablation inductively coupled plasma mass spectrometry (LA-ICP-MS) consisted of 40s background and 60s ablation data collection. All individual run data have been normalized to the mean of all NBS951 (0.0±0.4 ‰; 2se) data to allow for testing the reproducibility of both pelleted coral standards’ measurements (Jcp-1: 24.0±0.4 ‰; 2se). Note: no drift correction has been applied. Repeated measurements of glass standard NIST610 vs. NBS951 pellet yielded a mean δ^11^B for NIST610 of 0.3±0.2 ‰ (2se, n=160).


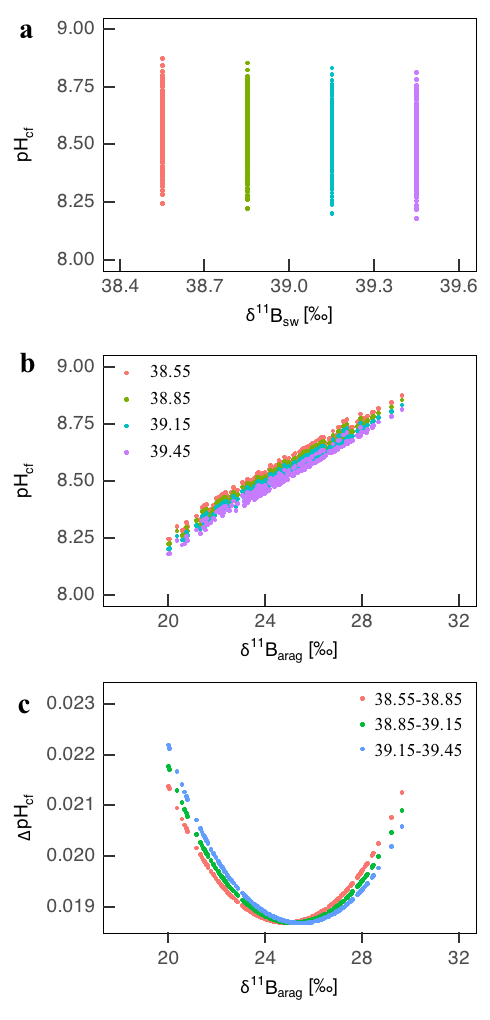


**Supplementary Figure 3:** **pH_cf_ systematics as a function of changes in seawater δ^11^B_sw_.** (a) pH_cf_ for the entire skeletal δ^11^B for different seawater δ^11^B_sw_. (b) pH_cf_ as a function of aragonite δ^11^B calculated from different δ^11^B_sw_ conditions and (c) how a change in seawater δ^11^B_sw_ changes pH_cf_ (∆pH_cf_) over the entire skeletal δ^11^B range.

**Supplementary Tables**

**Supplementary Table 1:** **Environmental conditions at the different sites.** Water chemistry measured (Salinity (S), Temperature (T), dissolved inorganic carbon (DIC), total alkalinity (TA)) and carbonate chemistry calculated (from dissolved inorganic carbon and total alkalinity for aragonite saturation state (Ω_arag_), pH_T_ (total scale), HCO_3_^-^ and salinity and temperature for pK_B_) from the control site (Co) and low pH centers (Ce) for the ojos in Puerto Morelos, Mexico: Norte (N), Agua (A) and Pargos (P); data are from Crook et al. ^2^.

| **Site** | **S (ppm)** | **T**  **(°C)** | **DIC**  **(µmol kg^-1^)** | **TA**  **(µmol kg^-1^)** | **Ω_arag_** | **pH_T_** | **HCO_3_^-^ (µmol kg^-1^)** | **pK_B_** |
| --- | --- | --- | --- | --- | --- | --- | --- | --- |
|  | measured values | | | | calculated values | | | |
| N.Ce | 33.8 | 28.7 | 2559 | 2601 | 1.22 | 7.41 | 2423 | 8.562 |
| N.Ce | 33.9 | 27.8 | 2409 | 2533 | 1.85 | 7.64 | 2261 | 8.572 |
| A.Ce | 33.1 | 27.6 | 2900 | 2997 | 1.82 | 7.56 | 2738 | 8.579 |
| A.Ce | 31.1 | 27.4 | 2904 | 2999 | 1.82 | 7.57 | 2743 | 8.594 |
| A.Ce | 32.7 | 27.5 | 3169 | 3096 | 0.77 | 7.30 | 2982 | 8.582 |
| N.Co | 35.1 | 29.2 | 2052 | 2399 | 4.03 | 8.04 | 1796 | 8.549 |
| N.Co | 35.3 | 29.4 | 2056 | 2404 | 4.03 | 8.04 | 1800 | 8.545 |
| N.Co | 35.3 | 29.4 | 2050 | 2406 | 4.12 | 8.05 | 1789 | 8.545 |
| A.Co | 34.8 | 28.8 | 2069 | 2398 | 3.83 | 8.02 | 1824 | 8.555 |
| P.Co | 35.4 | 28.8 | 2083 | 2392 | 3.60 | 8.00 | 1851 | 8.551 |
| P.Co | 35.3 | 28.2 | 2076 | 2387 | 3.60 | 8.00 | 1843 | 8.559 |
| P.Co | 34.9 | 28.6 | 2020 | 2388 | 4.24 | 8.09 | 1752 | 8.557 |

**Supplementary Table 2:** **Skeletal δ^11^B, B/Ca and derived calcification conditions for the different sites.** Skeletal **δ^11^**B and B/Ca and derived calcifying conditions: pH_cf_, pH-upregulation (∆pH), carbonate ion concentration (CO_3_^2-^_cf_), dissolved inorganic carbon concentration (DIC_cf_), DIC upregulation (DIC_cf_/DIC_sw_**)**, aragonite saturation state (Ω_cf_) and relative growth rate (G) (latter was calculated from both **δ^11^**B–derived pH_cf_ and B/Ca-derived DIC_cf_) for the different sites seawater aragonite saturation state (Ω_sw_). Min and max values for centres and control sites, Welchs t-test p-value comparing control and centres for each parameter and significance level: ns – not significant, * <0.05, ** <0.01. Values are mean ± sem.

| **Ω_sw centre & control_** | **δ^11^B** | | **pH_cf_** | | **∆pH** | | **B/Ca**  µmol/mol | | **CO_3_^2-^_cf_**  µmol/kg | | **DIC_cf_**  µmol/kg | | **DIC_cf_/DIC_sw_** | | **Ω_cf_** | | **G** | |
| --- | --- | --- | --- | --- | --- | --- | --- | --- | --- | --- | --- | --- | --- | --- | --- | --- | --- | --- |
| **0.77** | 24.8 | 0.4 | 8.53 | 0.03 | 1.23 | 0.03 | 679 | 22 | 768 | 31 | 2839 | 111 | 0.90 | 0.03 | 12.5 | 0.5 | 0.46 | 0.03 |
| **1.22** | 23.1 | 0.3 | 8.40 | 0.02 | 0.99 | 0.02 | 503 | 9 | 922 | 32 | 4028 | 80 | 1.57 | 0.03 | 15.0 | 0.5 | 0.80 | 0.06 |
| ***1.82*** | 23.2 | 0.4 | 8.44 | 0.02 | 0.88 | 0.02 | 529 | 14 | 808 | 22 | 3642 | 113 | 1.25 | 0.04 | 13.3 | 0.4 | 0.50 | 0.03 |
| **1.82** | 24.9 | 0.2 | 8.53 | 0.01 | 0.97 | 0.01 | 738 | 12 | 708 | 17 | 2587 | 48 | 0.89 | 0.02 | 11.5 | 0.2 | 0.39 | 0.02 |
| **1.85** | 23.2 | 0.3 | 8.41 | 0.02 | 0.78 | 0.02 | 569 | 11 | 819 | 25 | 3588 | 71 | 1.49 | 0.03 | 13.3 | 0.4 | 0.53 | 0.03 |
| **3.60** | 24.4 | 0.5 | 8.47 | 0.03 | 0.47 | 0.03 | 589 | 18 | 918 | 47 | 3474 | 133 | 1.67 | 0.06 | 14.8 | 0.8 | 0.80 | 0.09 |
| **3.60** | 24.2 | 0.4 | 8.45 | 0.02 | 0.45 | 0.02 | 568 | 20 | 940 | 26 | 3756 | 164 | 1.81 | 0.08 | 15.1 | 0.4 | 0.74 | 0.04 |
| **3.83** | 27.6 | 0.2 | 8.68 | 0.01 | 0.66 | 0.01 | 685 | 15 | 973 | 22 | 2666 | 58 | 1.29 | 0.03 | 15.8 | 0.4 | 0.89 | 0.04 |
| **4.03** | 25.4 | 0.5 | 8.53 | 0.03 | 0.49 | 0.03 | 598 | 14 | 965 | 42 | 3281 | 103 | 1.60 | 0.05 | 15.6 | 0.6 | 0.96 | 0.07 |
| ***4.04*** | 25.8 | 0.4 | 8.56 | 0.03 | 0.52 | 0.03 | 555 | 15 | 1087 | 39 | 3516 | 114 | 1.71 | 0.06 | 17.6 | 0.6 | 1.27 | 0.09 |
| **4.12** | 26.5 | 0.4 | 8.60 | 0.03 | 0.55 | 0.03 | 463 | 13 | 1350 | 56 | 4136 | 173 | 2.02 | 0.08 | 21.6 | 0.4 | 1.93 | 0.07 |
| **4.24** | 24.7 | 0.2 | 8.50 | 0.01 | 0.41 | 0.01 | 755 | 17 | 755 | 22 | 2757 | 72 | 1.36 | 0.04 | 12.1 | 0.4 | 0.51 | 0.03 |
| **Ojos centres** |  |  |  |  |  |  |  |  |  |  |  |  |  |  |  |  |  |  |
| **mean** | 23.8 | 0.41 | 8.46 | 0.03 | 0.97 | 0.08 | 604 | 45 | 805 | 35 | 3337 | 269 | 1.22 | 0.14 | 13.1 | 0.57 | 0.54 | 0.07 |
| **min** | 23.1 |  | 8.40 |  | 0.78 |  | 503 |  | 708 |  | 2587 |  | 0.89 |  | 11.5 |  | 0.39 |  |
| **max** | 24.9 |  | 8.53 |  | 1.23 |  | 738 |  | 922 |  | 4028 |  | 1.57 |  | 15.0 |  | 0.80 |  |
| **Ojos control** |  |  |  |  |  |  |  |  |  |  |  |  |  |  |  |  |  |  |
| **mean** | 25.5 | 0.5 | 8.54 | 0.03 | 0.50 | 0.01 | 601 | 36 | 998 | 70 | 3370 | 198 | 1.64 | 0.10 | 16.1 | 1.11 | 1.01 | 0.18 |
| **min** | 24.2 |  | 8.45 |  | 0.41 |  | 463 |  | 755 |  | 2666 |  | 1.29 |  | 12.1 |  | 0.51 |  |
| **max** | 27.6 |  | 8.68 |  | 0.66 |  | 755 |  | 1350 |  | 4136 |  | 2.02 |  | 21.6 |  | 1.93 |  |
| **Welchs t-test** |  |  |  |  |  |  |  |  |  |  |  |  |  |  |  |  |  |  |
| **p-value** | **0.022** |  | 0.085 |  | **0.002** |  | 0.860 |  | **0.036** |  | 0.850 |  | **0.045** |  | **0.042** |  | **0.036** |  |
| **significance** | ***** |  | ns |  | ****** |  | ns |  | ***** |  | ns |  | ***** |  | ***** |  | ***** |  |

**Supplementary References**

1. McCulloch, M., Falter, J., Trotter, J. & Montagna, P. Coral resilience to ocean acidification and global warming through pH up-regulation. *Nat. Clim. Chang.* **2,** 623–627 (2012).

2. Crook, E. D., Cohen, A. L., Rebolledo-vieyra, M., Hernandez, L. & Paytan, A. Reduced calcification and lack of acclimatization by coral colonies growing in areas of persistent natural acidification. *Proc. Natl. Acad. Sci. U. S. A.* **110,** 11044–11049 (2013).
